# Identification and analysis of alternative splicing events in *Phaseolus vulgaris* and *Glycine max*

**DOI:** 10.1186/s12864-017-4054-2

**Published:** 2017-08-22

**Authors:** Luis P. Iñiguez, Mario Ramírez, William B. Barbazuk, Georgina Hernández

**Affiliations:** 1 Centro de Ciencias Genómicas, Universidad Nacional Autónoma de México (UNAM), Cuernavaca, Morelos Mexico; 2 Department of Biology, University of Florida, Gainesville, FL USA

**Keywords:** Alternative splicing, Conservation of alternative splicing, RNA-seq, Legumes, Common bean, Soybean

## Abstract

**Background:**

The vast diversification of proteins in eukaryotic cells has been related with multiple transcript isoforms from a single gene that result in alternative splicing (AS) of primary transcripts. Analysis of RNA sequencing data from expressed sequence tags and next generation RNA sequencing has been crucial for AS identification and genome-wide AS studies. For the identification of AS events from the related legume species *Phaseolus vulgaris* and *Glycine max,* 157 and 88 publicly available RNA-seq libraries, respectively, were analyzed.

**Results:**

We identified 85,570 AS events from *P. vulgaris* in 72% of expressed genes and 134,316 AS events in 70% of expressed genes from *G. max*. These were categorized in seven AS event types with intron retention being the most abundant followed by alternative acceptor and alternative donor, representing ~75% of all AS events in both plants. Conservation of AS events in homologous genes between the two species was analyzed where an overrepresentation of AS affecting 5’UTR regions was observed for certain types of AS events. The conservation of AS events was experimentally validated for 8 selected genes, through RT-PCR analysis. The different types of AS events also varied by relative position in the genes. The results were consistent in both species.

**Conclusions:**

The identification and analysis of AS events are first steps to understand their biological relevance. The results presented here from two related legume species reveal high conservation, over ~15–20 MY of divergence, and may point to the biological relevance of AS.

**Electronic supplementary material:**

The online version of this article (doi:10.1186/s12864-017-4054-2) contains supplementary material, which is available to authorized users.

## Background

The majority of protein-coding genes from eukaryotic organisms contain introns, non-coding sequences that need to be spliced from the primary transcript to generate mature functional mRNAs. Although some introns can be self-spliced, most require a spliceosome, specialized splicing machinery. Spliceosomes are large ribonucleoprotein complexes that include small nuclear RNAs (snRNA) [1–3]. The spliceosome recognizes signals from common introns that allow their removal from the pre-mRNA. The U1 snRNA recognizes signals from the 5′ splice site, a GT dinucleotide. The U2 snRNA recognizes the 3′ splice site that includes an AG dinucleotide, an adenine which functions as a branching point upstream of the 3′ splicing site and a polypyrimidine tract between the branching point and the 3′ splicing site [4]. Other snRNAs, such as U11 and U12, recognize different splice sites, although spliceosomal introns of this class represent a minority [3, 4]. Different proteins that facilitate the recognition of the motifs by the spliceosome also mediate splicing. Serine/arginine-rich (SR) proteins facilitate the splicing of the intron while heterogeneous nuclear ribonucleoproteins inhibit the recognition of splicing sites [5]. The intron motifs as well as splicing enhancers and inhibitors are commonly found at different sites within the intron and these juxtaposed signals can give rise to variation in splicing an intron from the pre-mRNA; this phenomenon is known as alterative splicing (AS) [6–8].

AS is a post-transcriptional regulatory process that affects the fate of the mRNA; it has been found in several tissues, stress conditions and developmental stages of eukaryotic organisms [9]. AS can affect the localization of the mature mRNA and their translation efficiency [8]. Also, AS may produce alternative stop codons due to frame shifts in the mature mRNA sequence, thus regulating mRNA abundance by nonsense-mediated decay (NMD) [10]. Some other process linked to AS, such as mRNA storage or the target recognition of micro RNAs, have been reported [11, 12]. AS may also result in different protein isoforms derived from a single gene thus affecting protein localization or function [7, 13].

The evolutionary significance of AS has been related to organismal complexity. The number of genes in nematodes is very similar to that in humans although these organisms develop strikingly different cell types [14]. However, 98% of human multiple-exon genes undergo AS [15] in contrast to only 20% of nematode genes [16]. Chen et al. [17] analyzed several organisms that vary in their amount of different cell types –a proxy for organismal complexity- and found a strong positive correlation between the number of cell types and the level of AS. Organisms with higher complexity tend to have higher levels of AS.

AS genome-wide analyses, based on expression sequence tags (ESTs) and next generation RNA sequences (RNA-seq), have been reported for several plant species [18–27]. The first available resources for such analysis were EST databases that included sequences of large mRNA fragments often representing complete mRNA isoforms, but due to sampling the number of AS isoforms were likely underestimated. Next generation RNA-seq technologies produce a huge amount of sequences; however, these sequences are too short for complete isoform identification but can be used for characterization of AS events.

The recognition of splicing sites and the frequencies of AS types, vary between plants and animals. Animals tend to have very large introns and therefore the splicing machinery recognizes exons (exon definition) while in plants the spliceosome recognizes introns (intron definition) [28]. It has been proposed that a failure in the exon definition can lead to skipping an exon during splicing, while failure in intron definition results in intron retention [29]. The intron and exon definition models could explain the differences in the most common AS processes observed between plants and humans. Specifically, intron retention is the most abundant AS type observed in plants [30] while exon skipping is most common in animals [15].

Our research has focused on genome-wide analyses of transcriptional and post-transcriptional regulation in legume plants. Legumes are second only to Gramineae in their importance as crops; they are rich in protein content and have long been used for humans and animal consumption. Legumes are important contributors to biological nitrogen due to their ability to establish symbiosis with nitrogen-fixing soil bacteria (rhizobia). This relationship allows the legumes to grow under low or non-nitrogen fertilized media. Symbiotic nitrogen fixation has been a focus of research due to its economic and environmental importance [31]. Common bean (*Phaseolus vulgaris*) and soybean (*Glycine max*) are the most important legume crops worldwide. Common bean is the most important legume for human consumption as a source of proteins and micronutrients for millions of people, especially in Latin America and Africa where beans are important components of traditional diets [32]. Soybean is important worldwide as the dominant source of protein for animal feed and cooking oil [33]. These legumes are closely related and their evolutionary history makes them ideal models for genomic studies. Both the *P. vulgaris* and *G. max* genomes have been sequenced [33–35]. These legume species are evolutionary closely related, having diverged only ~19.2 million years ago (MYA), and share a whole-genome duplication (WGD) event ~56.5 MYA. *G. max* experienced an independent WGD ~10 MYA [34]. Thus, they are also good models to analyze features related to polyploidization.

This work analyzes AS in *P. vulgaris* and *G. max* by identifying and characterizing seven different AS events types genome-wide. This includes the identification of the introns/exons affected by AS and their relative position in the gene. AS event conservation between these legumes helps to elucidate some important aspects of the different types of AS. This work increases our knowledge of AS in legumes and provides a platform for further investigation.

## Results and discussion

### AS identification

**Fig. 1:**
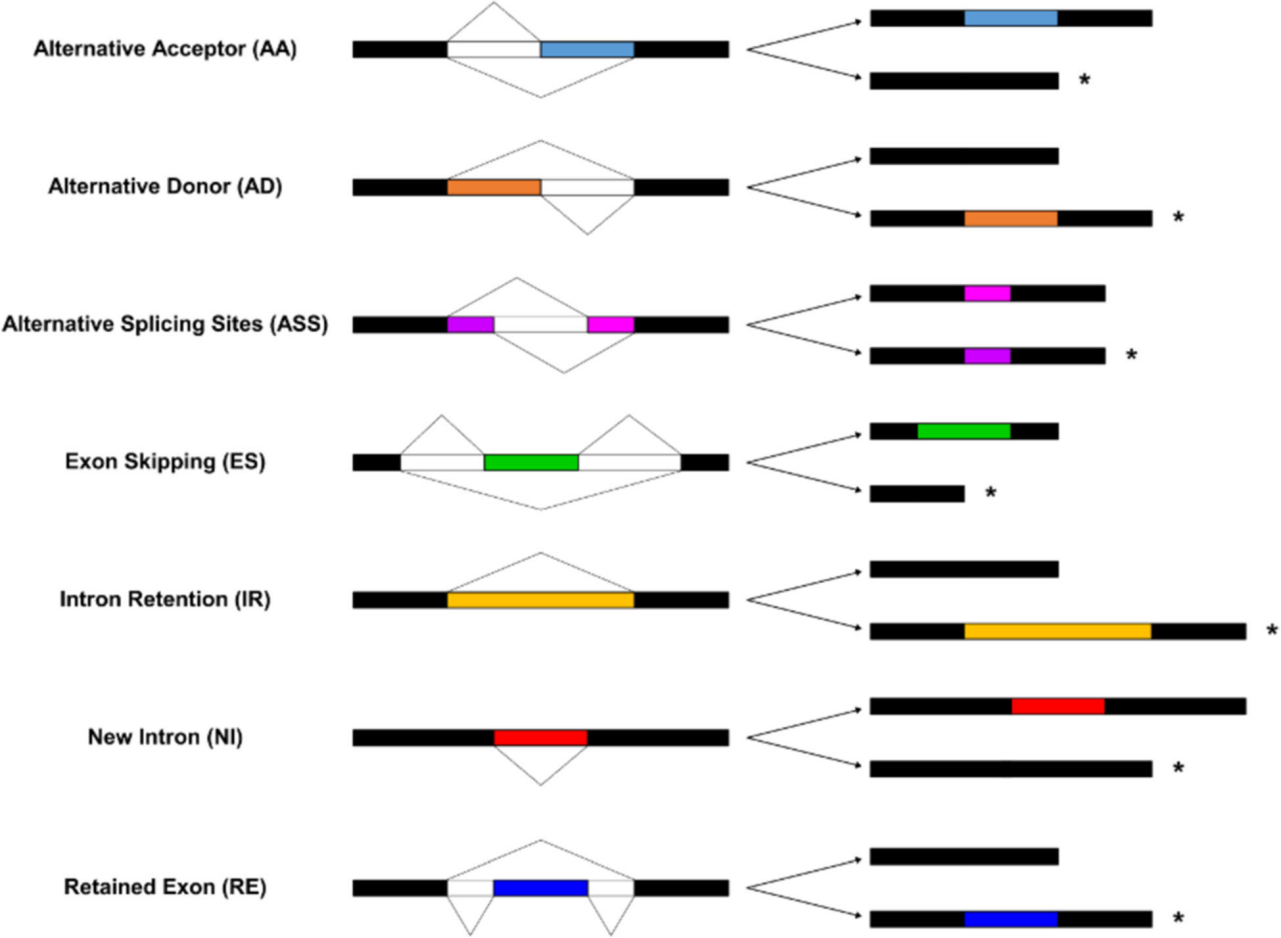
AS events. The types of AS events identified in this work are show, all of these are based on a primary transcript. Transcripts affected by AS are highlighted with an asterisk on the right side of the drawing. Drawing by Illustrator for Biological Sequences [66]

The characterization of AS events is a first step to understand the importance and prevalence of AS in plants. Four different types of AS events are the most frequently described in the literature: exon skipping (ES), where a whole exon is missed in comparison to the primary transcript; intron retention (IR), an intron is not spliced and is part of the mature mRNA; alternative donor (AD), the donor site, also known as 5′ splicing site, change in the mRNA isoform; and alternative acceptor (AA), where the 3′ splicing site is different. Based on a primary transcript three additional AS events can be described; alternative splicing sites (ASS), where both donor and acceptor sites change; new intron (NI), when a splicing site appears in a reported exon; and retained exon (RE), a new exon replaces a previously annotated intron in the mature mRNA. Schematic representations of these seven different type of AS events are presented in Fig. 1.

AS events were called when an exon-exon junction of a gene showed evidence for two or more splicing alternatives. The short length of RNA-seq sequences used for analysis allows the identification of AS events, but excludes the possibility of identifying whole isoforms; thus, alternative transcription start site, alternative polyadenylation site, translation start site and trans-splicing were not analysed.

Both the *P. vulgaris* and the *G. max* genome annotations [33, 34] include gene isoforms produced by AS in ~10 and ~23% of their genes, respectively. However, this is not consistent with reports that show higher proportions of AS genes in other plants [18–27, 36, 37]. The most frequent AS event reported for both legume genomes is AA, followed by AD and ES. In *P. vulgaris* the order of frequency following the first three events is IR, RE and NI and in *G. max* it is RE, NI and IR. The least frequent AS event reported for both plants is ASS (Additional file 1). Again, these results are not consistent with the AS profiles reported for other plants where IR the most common AS event, followed by AA and AD, and ES being the least common [7, 8, 36, 37]. These results suggest that the current annotation lacks a comprehensive identification of AS isoforms in these two legume species.

**Fig. 2:**
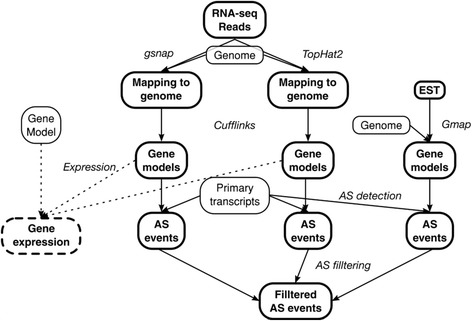
Method for AS event identification. Workflow for the identification of AS events in a single species. RNA-seq reads were mapped to their respective genome by two different algorithms, each mapping file was processed with Cufflinks to obtain the gene models. All gene models were compared to the primary transcripts to identify AS events. EST’s were also mapped and with both mapping files as well as the replicates all events were filtered. An expression value for each gene was calculated based on the mapping files

Here, RNA-seq and EST’s were analysed based on the methodology illustrated in Fig. 2, where two different mapping algorithms were used. The qualitative workflow allowed to identify AS events, based on the reported primary transcript. Thus AS events could be identified in any sample independently of the existence of the primary transcript in the same sample. This methodology (Fig. 2) led to the identification of AS events from *P. vulgaris* and *G. max* with a coverage of 88 and 72% of previously reported AS events*,* respectively. IR events were the most frequent for both species and ES the least frequent (Table 1). A total of 82,343 and 115,881 new AS events were identified for *P. vulgaris* and *G. max*, respectively (Table 1 and Additional file 2). The complete list of events covered 65% of *P. vulgaris* annotated genes and considering only the expressed genes included in the analysed RNA-seq samples (24,862), it was 72% of genes (Table 2). For *G. max*, 55% of annotated genes were affected by AS, or 70% of genes included in analysed RNA-seq libraries (43,712) (Table 2).

**Table 1:** Genome-wide AS identification

	*Phaseolus vulgaris*				*Glycine max*			
AS event	Identified from genome annotation^a^ [34]	New	Total	%	Identified from genome annotation^a^ [33]	New	Total	%
AA	1076	16,997	18,073	21	5209	26,901	32,110	24
AD	814	12,464	13,278	15	4126	20,113	24,239	18
ASS	59	6001	6060	7	343	5300	5643	4
ES	579	4285	4864	6	2543	8351	10,894	8
IR	387	33,091	33,478	39	1980	41,016	42,996	32
NI	115	3819	3934	5	2325	5267	7592	6
RE	377	5686	6063	7	1909	8933	10,842	8

^a^Total AS events reported in the annotated genomes are shown in Additional file 1

**Table 2:** Genes affected by AS

	*Phaseolus vulgaris*			*Glycine max*		
AS event	Number	Percentage		Number	Percentage	
		All	Expressed		All	Expressed
AA	8820	32%	35%	16,092	29%	37%
AD	7339	27%	30%	13,872	25%	32%
ASS	4117	15%	17%	3893	7%	9%
ES	3396	12%	14%	7431	13%	17%
IR	14,162	52%	57%	22,058	39%	50%
NI	3650	13%	15%	6990	12%	16%
RE	4940	18%	20%	8967	16%	21%
Total	17,789	65%	72%	30,677	55%	70%

Almost all (~98%) reported junctions in both legumes present the canonical intron motifs for the spliceosome recognition: the 5′ splicing site GT and the 3′ splicing site AG [4]. Most of the new junctions identified in this work, 78% for *P. vulgaris* and 85% for *G. max,* present the exact same motifs thus being considered substrates of the spliceosomal machinery (Additional file 3). The rest of the identified introns that presented non-canonical splicing sites, were also considered in our analysis based in previous knowledge about relevant regulatory roles of spliceosome-independent (self-splicing) introns from other organisms [38]. Nevertheless, the proportion of canonical splicing sites considered in this analysis –including reported and newly identified junctions- remains >93% for both legumes (Additional file 3).

The proportion of genes affected by AS are similar to those reported in other plants [18–27, 36, 37]. However, the distribution among types of AS events for both species differ from that reported in the genome annotations [33, 34] (Table 1 and Additional file 1). The three most frequent AS events identified for both plants were IR, AA, AD; corresponding to ~75% of all AS events (Table 1). Despite the different samples used for both species, the proportion of events as well as the number of genes affected by AS were similar (Tables 1 and 2). In mammals, the most common AS event is ES, ~50% of all events, this contrasts with plants where ES is generally less than 10% (Table 1) [9, 39]. Key processes have been implicated in the functionality of AS in plants for AA, AD and IR events. IR has been implicated in the process of NMD [10] due to the incorporation of stop codons. IR also plays an important role in *Mariselea vestita* in mRNA storage during it embryo development [12]. AA and AD are reported as consequences of small duplications in the splicing sites, allowing the incorporation or exclusion of one or several amino acids [40–43].

### Experimental validation of identified AS events

**Fig. 3:**
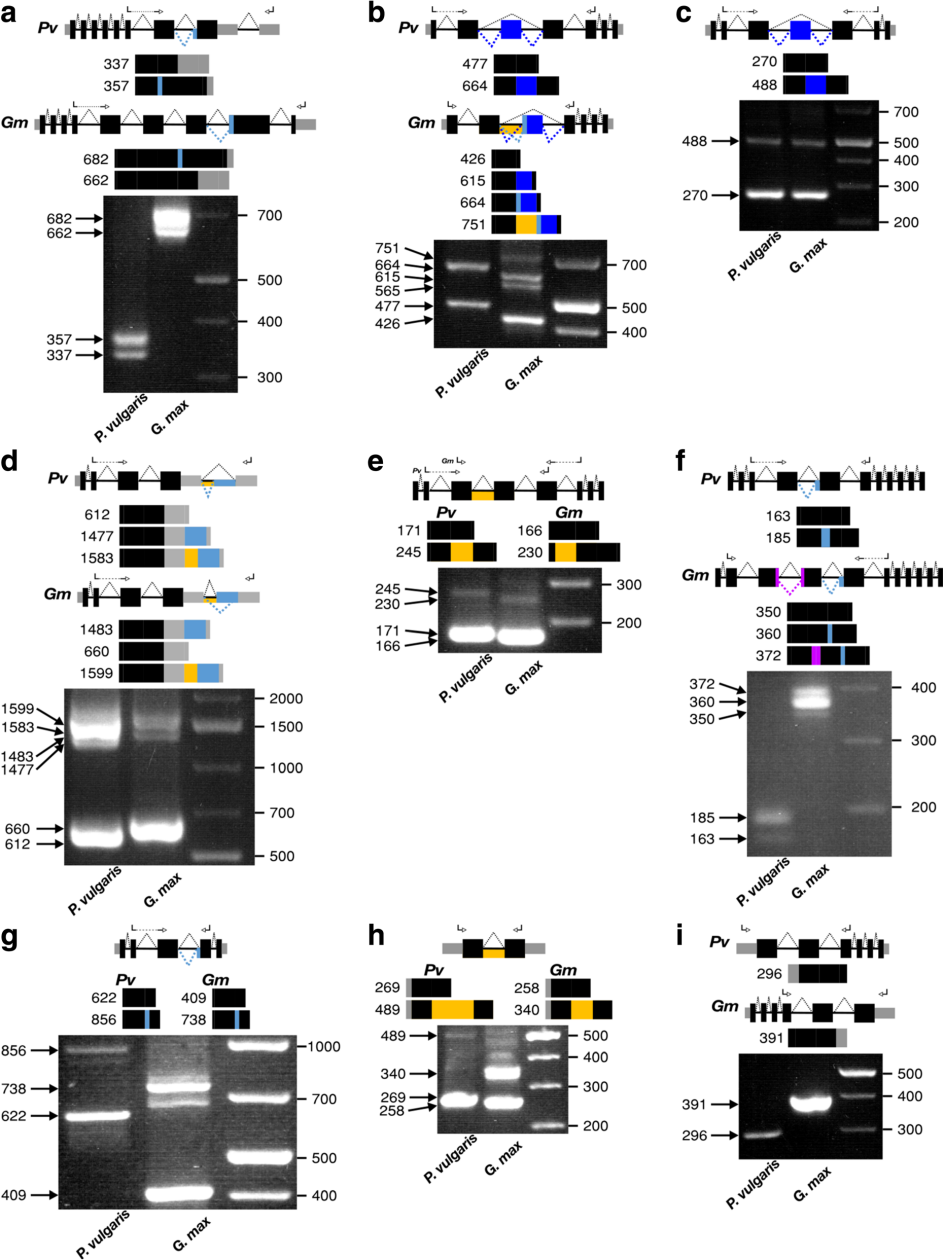
Experimental validation of AS events in *Phaseolus vulgaris* and *Glycine max* genes. Each selected gene, with conserved AS events in both species, is shown in a different panel: **a**) Phvul.011G190600, Glyma.13G187200; **b**) Phvul.008G270400, Glyma.02G293300; **c**) Phvul.007G262600, Glyma.09G104200; **d**) Phvul.005G127700, Glyma.12G181300; **e**) Phvul.003G077300, Glyma.07G259200; **f**) Phvul.002G298304, Glyma.08G023000; **g**) Phvul.001G014900, Glyma.14G075500; **h**) Phvul.006G191200; Glyma.13G245600; **i**) Phvul.008G013100, Glyma.18G289000. From top to bottom each panel includes: drawing of the gene model or of a different gene model for each species (not drawn to scale) with arrows indicating the position of the primers used for RT-PCR reactions and dotted lines indicating the splicing resulting in the primary transcript (above the gene model line) and the AS (below the line), the color code for different types of AS events is the same used in Fig. 1; drawings representing the amplification products expected for each transcript isoform, with its size (bp) indicated at the left; RT-PCR products resolved in 3% agarose gels, arrows indicate size (bp) of predicted fragments, the GeneRuler 1 kb Plus DNA ladder (Thermo Scientific, USA) was included for reference (third lane)

The RT-PCR approach was used to experimentally validate AS events selected from those identified in ~70% expressed genes from *P. vulgaris* and *G. max* genomes (Tables 1 and 2). Nine *P. vulgaris* genes and their corresponding *G. max* homolog, expressed in the plant tissues analyzed (roots and leaves from seedings), were selected for RT-PCR analysis. The selected genes presented conserved AS within both species (see subsequent sections). The two RT-PCR reactions performed for each gene, with RNA samples from root and from leaf, showed similar results in every case; Fig. 3 shows the results from leaves samples except for panels a and f that show results from root samples. In every gene analyzed the amplified products (ranging from 166 to 1599 bp) corresponded to expected fragments from the primary transcript or from transcript isoforms derived from AS events, according to each gene model (Fig. 3). The different types of AS events (Fig.1) validated for the selected genes included: AA (Fig. 3a–g), ASS (Fig. 3f), IR (Fig. 3b–h) and RE (Fig. 3b, c). As expected, only one amplified product corresponding to the primary transcript, could be observed in the control gene selected (Fig. 3i). In Fig. 3g, h additional amplification products, from those predicted from the gene models, could be observed; we cannot rule out that these correspond to AS events not identified in our analysis something that could be related to restrictions in the methodology we used. Taken together, the experimental results (Fig. 3) do validate and increase the reliability of the bionformatic data from this work.

### AS in CDS-UTR regions

The seven event types analysed here can be divided into two classes depending on how the AS event modifies the reference transcript. AA, AD, ASS, IR and RE events modify reference exon-exon junctions, while NI and ES alter reference exons, either excluding it or introducing a new intron (Fig. 1). There are three main types of introns defined by the untranslated regions (UTR) and coding DNA sequences (CDS) of the primary transcript, and six types of exons. The intron classification is based on the types of exon they are delimiting: 5’UTR-5’UTR, CDS-CDS and 3’UTR-3’UTR. Exons, on the other hand, can be classified in seven different types, 5’UTR, CDS, 3’UTR, 5’UTR-CDS, when the translation start site is in that exon, CDS-3’UTR, when the stop codon is in this exon, and 5’UTR-3’UTR which are genes without introns.

**Fig. 4:**
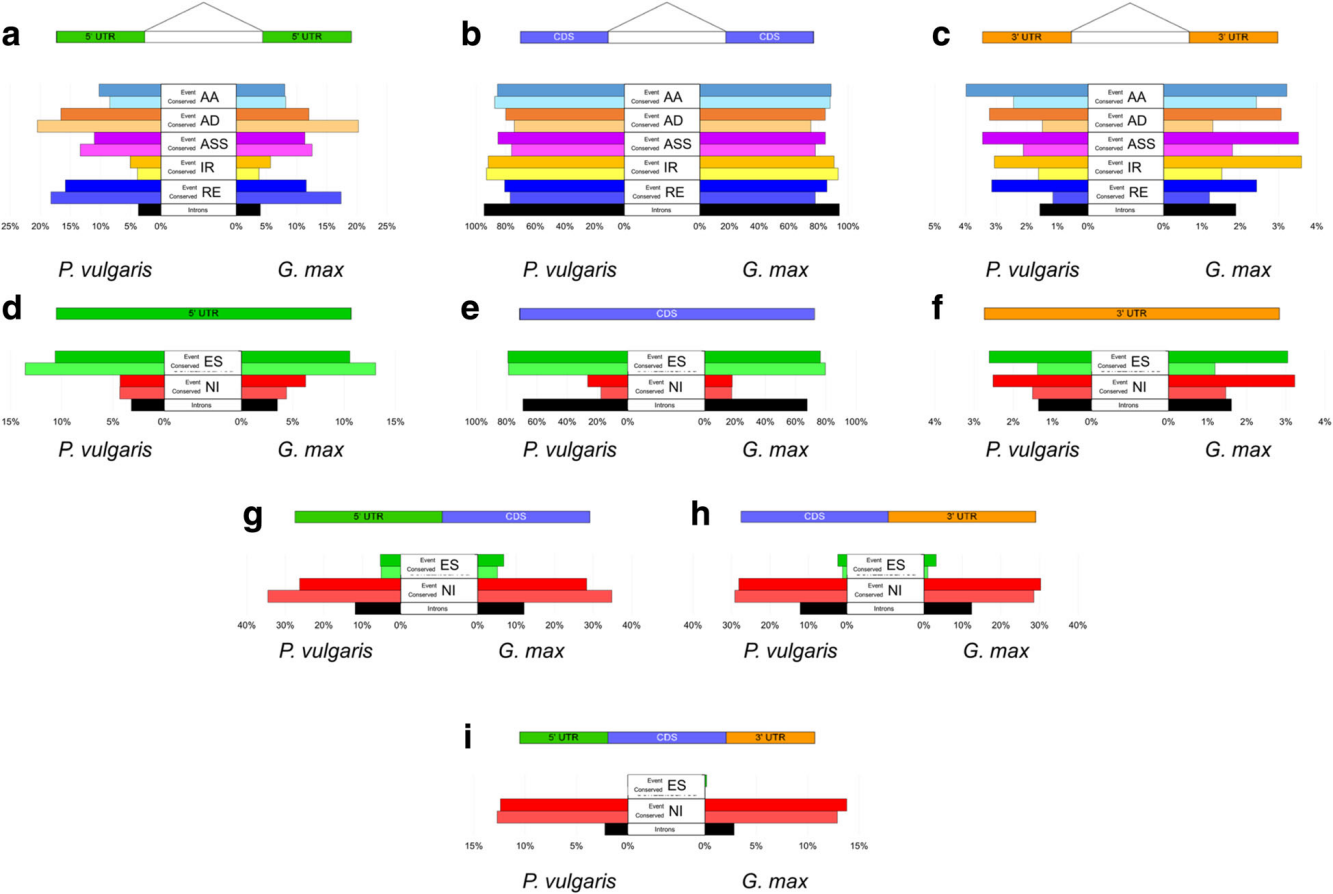
Percentage of introns and exons from UTR or CDS regions affected by AS events. AS events are dissected in terms of the percentage of exons or introns they affect. **a**, **b** and **c** show the introns affected by AA, AD, ASS, IR and RE (top to bottom, colored bars) and the percentage of introns in the genome (black bar, bottom). Each AS event includes two bar plots: the upper bar (dark tone color) shows the percentage of the type of junction is affected by AS event and the bottom bar (light tone color) shows the percentage of the type of junction affected in conserved AS events. The left side of each graph corresponds to *Phaseolus vulgaris* and the right side to *Glycine max*. **d**, **e**, **f**, **g**, **h**) and **i**) follow the same structure as the above, showing the percentage of exons affected by ES and NI

In both *P. vulgaris* and *G. max*, most introns of the primary transcript are CDS-CDS (~94%) and the rest are 5’UTR-5’UTR (~4%) and 3’UTR-3’UTR (~2%) (Fig. 4a-c). In the case of exons, also in both genomes, the majority (69%) were CDS (Fig. 4e), while the other exon types, 5’UTR-CDS and CDS-3’UTR, correspond to 12% (Fig. 4g, h). The other 7% of exons were divided into 3% single exon genes (Fig. 4i), 3% 5’UTR and 1% 3’UTR exons (Fig. 4d, f).

The introns, or exon junctions, affected by the AS were analysed to determine if they were randomly distributed. The percentage of each type of intron/exon in the genome was compared to the percentage affected by AS. Both legumes showed an enrichment of AS events in UTR introns (Additional file 4), whereas, AS events in CDS junctions were under-represented, despite being the majority of affected junctions. Similar data were observed for each individual AS event (Fig. 4a-c). In the case of the exons affected by AS a decrease of CDS exons and an over-representation of every other type of exon were observed (Additional file 4). Interestingly in the case of exons, the under- and over-representation of each type of AS event varied among types of exons. CDS exons were underrepresented in NI events while overrepresented in ES events (Fig. 4e). In contrast, 5’UTR-CDS and CDS-3’UTR were enriched in NI and decreased in ES events (Fig. 4g, h). Common bean and soybean showed similar results (Fig. 4).

While the main effect of ES is to skip CDS exons, NI introduces introns in combined exons such as 5’UTR-CDS or CDS-3’UTR. Marquez et al. [44] reported the presence of NI in single exon genes and called them “exitrons”. Together, these results point to a non-random distribution of AS and to the selection of AS in specific regions of the genes. The similar results obtained for both legumes suggest important aspects of specific AS events in these species.

An interesting example of AS in CDS-CDS regions was identified in the CSN7 gene, this protein is one of the eight subunits of the COP9 signalosome (CSN) that is a key player in the DNA-damage response, cell-cycle control and gene expression. CSN7, as well as other 5 subunits, contains a N-terminal PCI domain that is important for subunits interactions. In addition, CSN7 C-terminal tail is responsible for interactions with the non-PCI protein CSN6 as well as with other proteins such as the ribonucleotide reductase RNR2. The different protein-protein interactions of CSN7 C-terminal regulate the CSN complex assembly as well as the function of RNR2 [45]. As shown in Fig. 3a, the CSN7 gene from *P. vulgaris* (Phvul.011G190600) and *G. max* (Glyma.13G187200) presents a conserved a AS event, of the AA type, in a CDS-CDS junction (intron 7) of the C-terminal region. The reported primary transcript differs among these species, being the *P. vulgaris* primary transcript similar to an alternative transcript isoform of *G. max* and viceversa (Fig. 3a). The AA transcript isoform from *P. vulgaris* presents a modification of the reading frame from exon 8 that shifts the stop codon to exon 9 (Fig. 3a). In *G. max* CDN7 the stop codon of the primary transcript is located in exon 9 and a similar AA event is present on intron 7 with the stop codon in exon 8 (Fig. 3a). This AA event on both species modifies the C-terminal of CSN7, we propose this could regulate the interactions with different proteins thus affecting the functionality of CNS in these legume species.

### Conservation of AS between *P. vulgaris* and *G. max*

Different approaches may be used to analyse the conservation of AS between homologous genes. Here, we used a junction conservation approach rather than the overly strict position conservation approach. The position conservation approach is based on the conservation of the event in exact positions while for junction conservation only the event and intron must be conserved [46]. An AS event was considered conserved if both homologous genes from *P. vulgaris* and *G. max* showed the same AS event at a specific junction. Redundant AS events were removed from identified AS events in both plants. Redundant AS events imply that the same type of event occurred in the same intron or exon but at a different position. Since a junction conservation approach was used for the AS event conservation analysis the exact position of the AS event was, for this study, irrelevant.

**Fig. 5:**
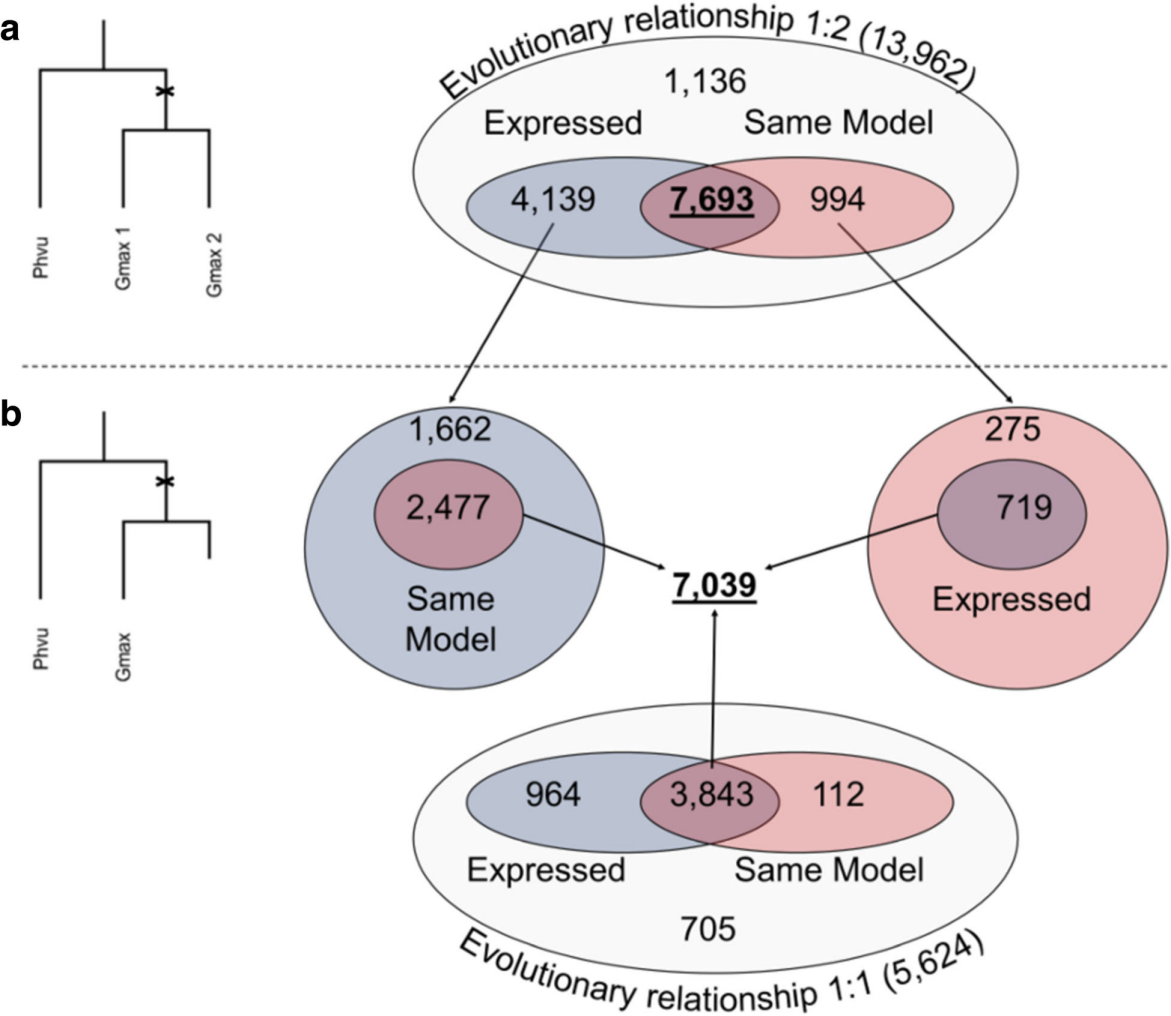
Homologous *Phaseolus vulgaris* and *Glycine max* genes. The left side of each panel is a schematic representation of the evolutionary history of a gene, the cross represents the WGD that occurred in *G. max* after the speciation of both plants. **a** Number of *P. vulgaris* genes with two copies in *G. max* (1:2) **b** the number of *P. vulgaris* genes with one copy in *G. max* (1:1). Different color-coded ellipses indicate the numbers of expressed genes, genes with the same gene model between both species and genes sharing these the two characteristics. The number of *P. vulgaris* genes for AS conservation analysis are underlined

The first step to identify the conservation of AS events was to define *P. vulgaris* and *G. max* homologous genes with the same gene model. *P. vulgaris* and *G. max* have a relative short evolutionary distance of ~19.2 MY [34] and soybean experienced a recent WGD, ~10 MYA; therefore, a high proportion of common bean genes (51%) have two homologous genes in soybean (representing 50% of the total gene set), resulting in a 1:2 relationship. As shown in Fig. 5a, 13,962 *P. vulgaris* genes with 2 *G. max *homologs were identified. Of these 55% (7693) were expressed and had the same gene model in both *G. max* homologs. There were 7039 *P. vulgaris* genes with only one identifiable homolog in *G. max* (Fig. 5b) (including genes with a 1:2 relationship but where one did have the same gene structure or only 1 *G. max* homolog was expressed, plus those genes with only one expressed homolog with the same gene structure). In total 14,712 *P. vulgaris* genes and 22,405 *G. max* genes, representing more than 50% of expressed genes in both plants, were selected for analysis of AS conservation.

The junction conservation approach considers the type of AS event and the affected intron/exon. Therefore, if two events from the same type that coincide in a single intron/exon, even although they differ in positions, were considered redundant and were collapsed into non-redundant events. 61 and 51% of all non-redundant events belong to the genes described in Fig. 5 in *Phaseolus vulgaris* and *Glycine max*, respectively. The proportions of the types of AS in the non-redundant events is similar in genes with homology in the other species relative to the proportions of AS types in all expressed genes (Additional file 5).

**Fig. 6:**
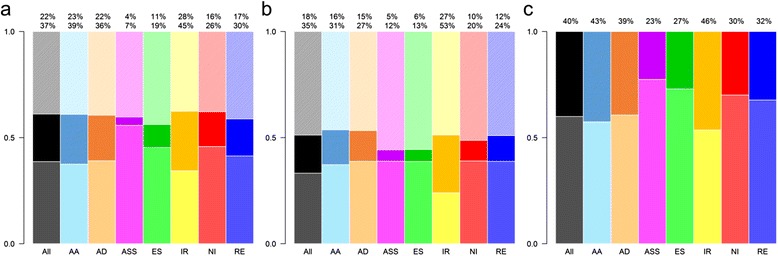
Conservation of AS among *Phaseolus vulgaris* and *Glycine max.* Cumulative bar plots represent total AS events (black bars) and each AS event type (colored bars). In every bar plot the stripped bar (top) corresponds to AS events in non-homologous genes, the dark tone color bar (middle or top) to conserved AS events and the light tone color bar (bottom) to non-conserved AS events in homologous genes. The numbers over the bar indicate the percentage of conserved events over all AS events (top) and over AS events in homologous genes (bottom). AS event conservation of *P. vulgaris* in *G. max* (**a**), of *G. max* in *P. vulgaris* (**b**) and in paralogous *G. max* genes, originated by its recent WGD (**c**)

Considering only homologous genes with the same model between *P. vulgaris* and *G. max* the rate of conservation of *P. vulgaris* AS events within *G. max* was 37%, here assumed as the maximum conservation rate. Since not all the homologous genes were considered, the minimum rate of AS conservation was 22% based on all non-redundant events in common bean (78,027) (Fig. 6a). On the other hand, the rate of AS conservation of *G. max* in *P. vulgaris* homologs with the same gene model was 35 and 18% of AS events for all soybean non-redundant events (121,133) (Fig. 6b). Interestingly, conservation differed depending on the type of AS event. The proportion of IR events from *P. vulgaris* conserved in *G. max* was 45 (Fig. 6a) and 53% of the soybean IR events were conserved in common bean homologous genes (Fig. 6b). Following the order of conservations, AA and AD stand after IR (Fig. 6a, b), coinciding with the order of abundance of AS types (Table 1).

These results support the suitability of using the junction approach for identifying AS conservation among homologous genes. Chamala et al. [46] reported that the proportion of conservation of AS events varies among the types of AS events. They analysed four types of events (AA, AD, ES and IR) and found IR to be the most conserved type, consistent with our data. The results of Chamala et al. [46] and this work indicate that IR, AA and AD are not only the most common events in both legume plants but these present the highest AS event conservation rate across angiosperms. Their evolutionary conservation indicates potential function. Although ASS and ES as well as NI and RE have similar proportions in both species, the first two are less conserved. This could be interpreted as ASS and ES being more species specific than NI and RE or their function is not conserved.

The data used for these analyses also enabled the identification of AS events conserved between soybean paralogs that arose during the last WGD. The paralogous genes set must have identical gene models in order to identify the conserved events by junction. A total of 40% AS events were conserved among *G. max* paralogous genes (Fig. 6c). Similar as seen for AS conservation between species, IR was the type of AS event with the highest conservation rate followed by AA and AD; while AS and ES showed the lowest percentage of conservation although this was higher than 20% (Fig. 6c). New AS events could arise after the WGD within either one or both paralogs, isoforms could have predated the WGD and still remain in both paralogs, or only one of the paralogs could have lost its isoform subsequent to the WGD event. To address this question further analyses on conserved AS within genes duplicated through WGD in another species need to be examined.

The conservation of AS events between these two legumes may be the result of their performing an essential function, particularly since these events have been conserved over ~20 MY. The isoforms produced due to AS could be important for a specific tissue, condition or developmental stage. To understand the function, and/or temporal and spatial conditions under which these AS isoforms are expressed will require additional investigation. However, since the majority of the AS events are not conserved, the lack of conservation of AS events may not only be due to their biological function, but may reflect the divergence time between these species. Paralogous genes in *G. max* with shorter diversification time accounted for a majority of the gene specific AS events. A combination of functionality and diversification time could lead to the percentage of AS conservation observed here.

Two examples of AS events conserved among plant species that indicate their important biological functions, are the following. The transcription factor TFIIIA is required for the synthesis of 5S–rRNA by RNA polymerase III. The third exon of this gene, with a structural element that mimics 5S rRNA, presents an ER event [47–49]. This TFIIIA exon, that is highly conserved in land plants [50], is referred to as a suicide exon because its absence produces a functional transcript whereas its retention results in a non-functional transcript containing premature termination codons (PTC) thus targeted to NMD. The L5 ribosomal protein binds to the 5S rRNA mimic and controls the synthesis of 5S RNA by regulating AS of TFIIIA [48]. Fig. 3b shows that the ER event identified in the third exon of Arabidopsis TFIIIA is conserved in *P. vulgaris* (Phvul.008G270400) and *G. max* (Glyma.02G293300). The absence or presence of the exon is clearly observed in both species and *G. max* presents additional transcript isoforms varying in the length of the RE (Fig. 3b). As in Arabidopsis, PTC were identified in the RE in both legume species, thus indicating the conservation of AS and of regulation/function of TFIIIA in the legume species analysed in this work.

Another example is the SCL33, a protein from the SR family that regulate splicing by binding to splicing regulatory elements -found in exons or introns-, facilitating spliceosome assembly and enhancing splicing [51]. The extensive AS of the third intron of Arabidopsis SCL33, that includes a RE with PTC, results in potential targets of NMD and these have been implicated in auto-regulation of AS by SCL33 [52]. This AS event is conserved in other plants such as *Brachypodium distachyon* [27]. Fig. 3c shows that an RE event in the third intron is also conserved in *P. vulgaris* (Phvul.007G262600) and *G.max* (Glyma.09G104200) SCL33; the RE presents PTC in both legume species. We propose that the AS-related function of the legumes’ SCL33 gene is similar to that known for Arabidopsis, regarding its targeting to NMD and the auto-regulation of SCL33 protein content.

### Conservation of AS in UTR-CDS regions

Conserved AS events between *P. vulgaris* and *G. max* constitute a small proportion relative to all AS events in each species (Fig. 6). However, an assumption we made was that conserved AS events may have a biological function. The percentage of intron/exons of UTR and CDS regions affected in conserved AS events from homologous genes as compared to all AS events were analyzed to explore potential function (Fig. 4).

Regarding AS events in 5’UTR-5’UTR junctions (Fig. 4a), AD, ASS and RE showed a higher percentage of conserved events as compared to all AS events in both plants. These data indicate that AD, ASS and RE events are preferentially conserved in 5’UTR introns, which could imply a conserved function, though such function is yet unknown. AS occurring in 5’UTR regions has been implicated in upstream open reading frames (uORF) of small proteins, that in turn have been implicated in the mRNA stability through NMD and in translation efficiency [53]. Nevertheless, the amino acid conservation among uORF from different organisms suggests a possible translation of small proteins and a possible function of these [53]. Proteomic studies would be required for further studies on the existence and function of such small proteins in legumes.

IR, however, exhibits a different pattern since the percentage of conserved events in 5’UTR-5’UTR junctions was lower than the percentage of all IR events (Fig. 4a). Nevertheless, in both species the percentage of IR was higher than the percentage of all events from CDS-CDS junctions (Fig. 4b), indicating a possible role for this type of event in CDS introns. In contrast, a reduction in the percentage of conservation of AD, ASS and RE in CDS introns was observed in both plants (Fig. 4b). For AA the percentage of conserved events in the 5’UTR and CDS introns followed a different pattern between *P. vulgaris* and *G. max* (Fig. 4a, b), showing that 5’UTR or CDS regions do not affect clearly the conservation of AA events. Interestingly these five types of AS events that affect introns showed a reduction in percentages of 3’UTR-3’UTR junctions in conserved AS events as compared to all events in both species (Fig. 4c).

A similar analysis was done for ES and NI events that affect exons (Fig. 4d-i). Despite the differences in conserved NI event proportions between these two species, this AS was enriched in conserved AS events, for 5’UTR-CDS, CDS-3’UTR exons and single exon genes (Fig. 4g–i). This indicates potential functional relevance in those regions. Notably, the 3’UTR exons showed a reduction in the percentage of conserved events for ES and NI compared to all events as well as for the five AS events affecting introns (Fig. 4f).

Taken together the results of conserved AS events in CDS and UTR regions suggest that the potential of AS to affect either introns or exons is greater on regions upstream from the 3’UTR region.

An example of conserved AS in the 3’UTR region was identified for U2AF35, that is a component of the U2AF (U2 snRNP auxiliary factor) heterodimer, an essential pre-mRNA splicing factor. U2AF35 plays critical roles in the recognition of the 3′-splicing [54]. In addition human U2AF35 is implicated in the determination of mRNAs 3’UTR-length; mutated U2AF35 results in longer 3’UTR of certain genes [55]. AS in 3’UTR has been associated with the regulation of protein expression, by yet unidentified mechanisms [56]. Figure 3d shows the conserved 3’UTR AA event in *P. vulgaris* (Phvul.005G127700) and *G. max* (Glyma.12G181300) resulting in mature U2AF35 mRNAs varying in their 3’UTR length (Fig. 3d). This is another example of a gene with different primary transcript in both species; the primary transcript of one is similar to the alternative transcript isoform of the other (Fig. 3a, d). Additionally, a conserved IR event was validated in both species (Fig. 3d). Based in previous knowledge [55], we hypothesize that protein expression of U2AF35a in both legume species could be self-regulated through AS.

### AS simulation

An important question in this work was if the 22 and 18% of AS conservation (Fig. 6) is a significant result from the *P. vulgaris* and *G. max* comparison, or if such values are just been obtained by chance. To answer this question four different simulations for AS event conservation were performed (Additional file 6). In the first simulation (“random events”) all the non-redundant AS events in the expressed genes were shuffled, thus any intron/exon could be alternative spliced and the real homologous genes were maintained. In the second simulation (“random genes”) the genes were shuffled to consider different homologs with their AS events maintained, the distribution of the number of exons per gene (Additional file 7) was not considered. The third simulation (“random genes same model”) was like the second, maintaining real AS events, but the exon distribution was that of the real homologs (Additional file 7). The objective of “random genes” and “random genes same model” simulations was to explore if the homology between *P. vulgaris* and *G. max* genes was important for the AS event conservation. The last simulation (“random genes + random event”) where homologous genes and AS events were shuffled was a combination of the first and second simulations (Additional file 6).

**Fig. 7:**
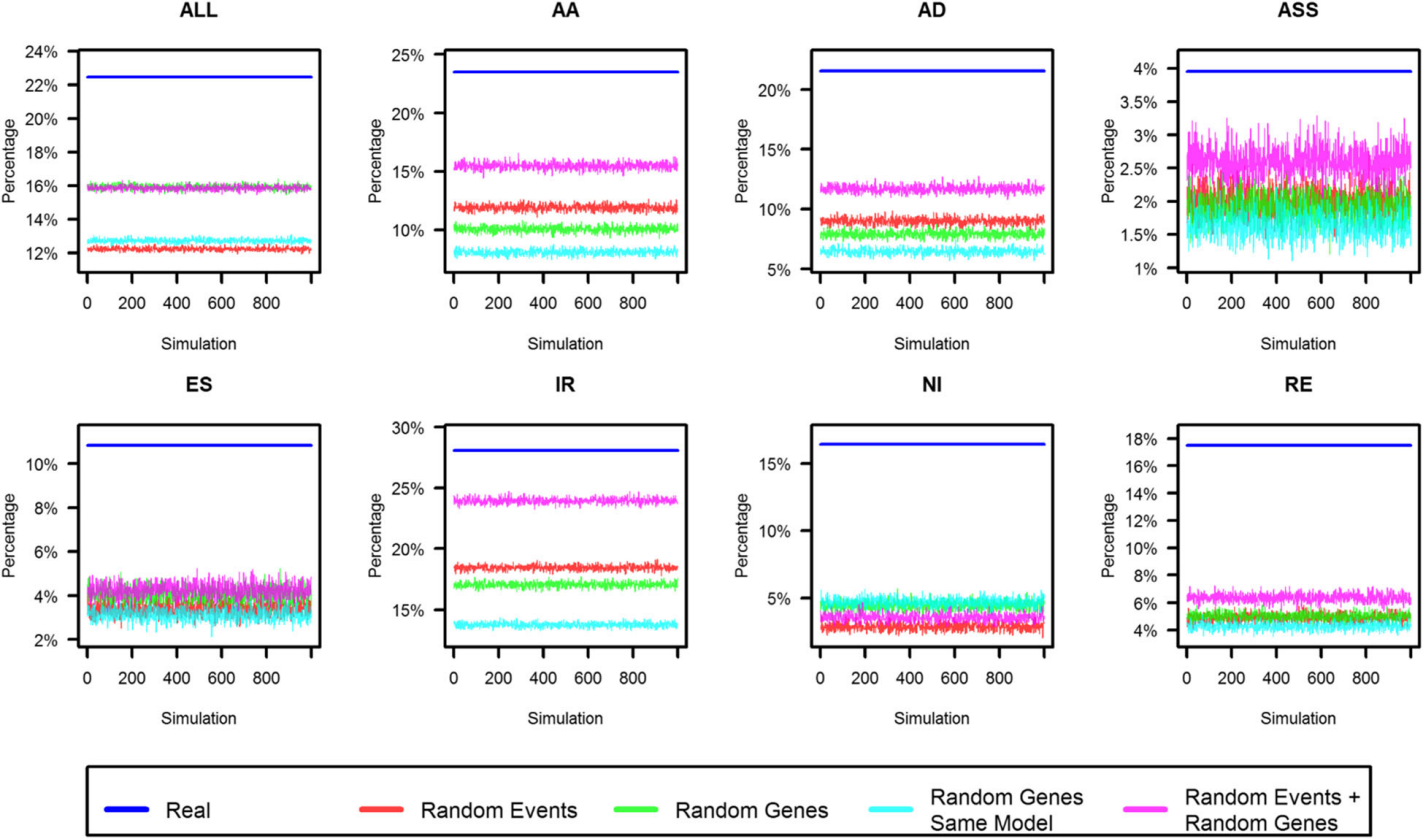
Comparison of conservation percentages of AS events in *Phaseolus vulgaris.* Four different simulations were performed; “random events”, “random genes”, “random genes same model” and “random genes + random events”, each one is represented with a different color as indicated. The blue line corresponds to the values of percentages of AS conservation over all AS events of *P. vulgaris* in *G. max* (shown in Fig. 5a). Conservation of all AS events and of each type of AS events was plotted individually, as indicated. For each type of simulations, 1000 randomizations were preformed and the percentage of conservations for every simulation was plotted

The data on percentages of AS conservation (Fig. 6) were compared to the data obtained for each of the simulations; data of *P. vulgaris* in *G. max* are represented in Fig. 7 and *G. max* in *P. vulgaris* in Additional file 8. All simulations resulted in a value for the percentage of AS conservation that is lower than observed in our analysis for both overall and individual AS events, thus indicating that these results are neither random or artefacts (Fig. 6, Additional file 8).

The analysis of conservation of each type of AS derived from the simulations revealed interesting features such as a correlation of exon number with the conservation of AS events. For every type of AS event, except NI, the “random genes + random event” simulation showed the highest percentage of conserved AS (Fig. 7). One interpretation is that having more exons increases the probability of having AS and therefore the AS event conservation is also more likely. NI does not follow this rule as gene structure plays a major role for this type of AS event. This is consistent with the results of NI percentage in the UTR-CDS regions, where a high percentage was observed in the single exon genes (Fig. 4i), similar to a report by Marquez et al. [44]. For NI the highest percentage of conservation was observed in “random genes” and “random genes same model” simulations (Fig. 7). This suggests that higher NI occurrence is also related to specific genes.

The simulations also provided insights into the conservation of AS events. The position of the AS events within the gene tends to influence AS event conservation. If the positions of the AS conserved events within the gene were random, the percentage of conservation from “random events” and “random genes same model” would be similar. However, “random events” presented higher percentage of conservation in AA, AD and IR events (Fig. 7), something that could indicate that some homologous genes tend to present AS event conservation in specific introns or exons. The most common AS event types (AA, AD and IR) showed a bias for conserving AS events relative to position within the gene, as well as the number of exons, as described above. This tendency added to the exon number tendency were observed in the “random genes same model” simulation with the lowest conservation of AS events in AA, AD and IR (Fig. 7). A similar phenomena was observed for “random genes” in comparison with “random genes + random events” simulations, where besides having more exons the percentage of AS event conservation was lower in “random gene” simulation than when randomizing events (“random events + random genes”) (Fig. 7, Additional file 7). This indicates that there is a bias for certain genes to conserve these types of events in certain introns. With these results, it is not possible to determine if RE, ASS and ES types have a bias in the position, as the percentages of conserved AS events in the simulations were similar due to the low number of non-redundant AS events (Fig. 7 and Additional file 8).

### AS position within the gene

**Fig. 8:**
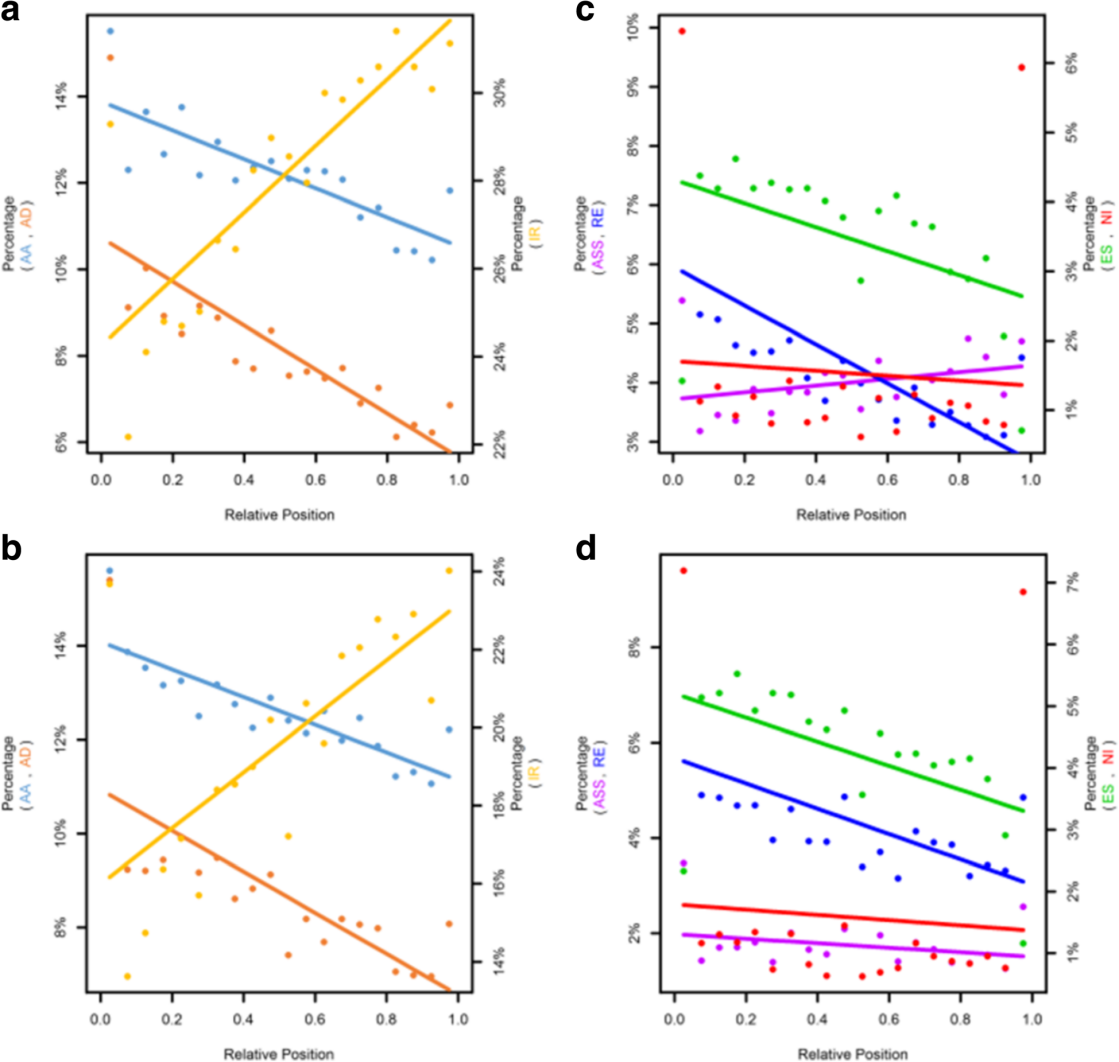
Linear modeling of AS events relative to the position of affected exon/intron. Each event was characterized to their relative exon/intron of the gene affected, being 0 the first or only intron/exon and 1 the last. Each type of AS event is presented separately. A simple linear model was fitted to the data. **a** distribution of AA (r^2^ = 0.63 *p*-value = 1.418e-05), AD (r^2^ = 0.6 *p*-value = 3.358e-05) and IR (r^2^ = 0.67 *p*-value = 5.656e-06) for *P. vulgaris* the left axis represent the percentage of AA and AD and the right one of IR; **c** Distribution of ASS (r^2^ = 0.05 *p*-value = 0.1769), RE (r^2^ = 0.64 *p*-value = 1.418e-05), ES (r^2^ = 0.19 *p*-value = 0.02895) and NI (r^2^ = 0.05 *p*-value = 0.7848), the left axis is for the intron events (ASS and RE) and the right axis for the exons affected (ES and NI) for *P. vulgaris*. **b** and **d** are same as (**a**) and (**c**) but for *G. max*. (AA: r^2^ = 0.71 *p*-value = 2.216e-06, AD: r^2^ = 0.49 *p*-value = 0.0003575, IR: r^2^ = 0.45 *p*-value = 0.0006914, ASS: r^2^ = 0.02 *p*-value = 0.256, RE: r^2^ = 0.29 *p*-value = 0.008569, ES: r^2^ = 0.24 *p*-value = 0.01595, NI: r^2^ = 0.05 *p*-value = 0.7779)

For an in-depth analysis of the position of AS event within a gene the affected introns/exons were catalogued with respect to their relative position in the gene, with the first or single exon/intron designated as 0, and the terminal exon designated as 1. The percentages of affected exons/introns for each relative position were calculated (Fig. 8). A linear regression was calculated for each type of event looking for a bias in the positions of AS events within the genes. In agreement with the results from the simulations, AA, AD and IR presented a positional bias (Figs. 7, 8). AA and AD were enriched in initial introns, in contrast to IR where there was a bias for the terminal introns (Fig. 8a, b). RE and ES did not show a clearly position bias in the conservation studies, while this analysis uncovers a tendency of these events to affect initial introns in both plants (Fig. 8c, d). This position preference was similar to that seen for AA and AD. These results were consistent in both species (Fig. 8) and highlight the importance of the position of the AS event. The position bias of AS events within the gene observed for different AS events provide insights into how and in which gene positions each AS event is regulated.

It has been observed that the splicing occurs co-transcriptionally and sometimes it depends on the rate of the RNA-polymerase reaction [57]. The transcription rate also plays an important role in the formation of the secondary structure of the nascent RNA. The secondary RNA structure has been implicated in AS, there are some structures that prevent splicing site recognition and others that facilitate it [58]. The inhibition of splicing due to secondary RNA structure has been related with a competition between the structure and the site recognition of splicing factors. Therefore the splicing efficiency is directly correlated with the stability of the secondary RNA structure. On the other hand RNA structures could bring important splicing signals into closer proximity enhancing the splicing [59]. Based on this information we hypothesize that at transcription initiation, the nascent RNA still lacks a stable secondary structure thus facilitating the splicing site recognition and producing AS events such as AA, AD, ES or RE. IR events presented a tendency for terminal introns from already transcribed RNA with defined secondary RNA structures that could inhibit the recognition of splicing sites thus resulting in an IR. Nevertheless, these hypotheses need to be tested in order to better understand the regulation of the different AS events.

## Conclusions

The algorithms and methodology used in this work allowed the identification and analysis of seven different types of AS events from two agronomically important legume species: *P. vulgaris* (common bean) and *G. max* (soybean). While the number of AS events was highly underestimated in their respective genome sequence annotations [33, 34], here it was shown that ~60% of all genes and ~70% of expressed genes from both species may undergo AS. Each type of AS event affected a different proportion of genes, with IR being the most frequent AS event followed by AA and AD in both plants.

The AS events were characterized in terms of the region they affected, and their relative position within the gene. The results of this characterization exposed different patterns for each of the AS events, such as preference for single exon genes from NI events or the contrasting result for position preferences between AA, AD and IR. These results were similar for both species, highlighting global aspects of these AS events in these legumes.

The conservation of AS events in two evolutionary related legume species was analysed considering those *P. vulgaris* genes with two (evolutionary relationship 1:2) or one (1:1) homologous gene in *G. max*. A significant proportion (ranging from 18 to 37%) of AS events were conserved between species. The conserved AS events are key to further research since they have been conserved for ~20 MY and they may provide insights into the functional role of these AS events in legumes. The conservation of AS events was experimentally validated for 8 selected genes, through RT-PCR analysis, something that enhances the reliability of bioinfomatic data from this work. The proposed function and biological significance of some of the validated conserved AS events was discussed, nevertheless they need to be further studied. The percentage of AS event conservation varies among the different AS types, with IR, AA and AD the most highly conserved in both species. Conserved events were also characterized in terms of the gene region they affect. The results threw a significant tendency to conserve events upstream 3’UTR regions, which indicates that events in 3’UTR are preferentially specie specific.

This work increases the knowledge of the yet almost unexplored process of AS. Tissue specific analysis, as well as isoform analyses, need to be performed to understand this relevant process for genome expression/function in eukaryotes.

## Methods

### RNA-seq libraries and expressed sequenced tags (ESTs)

All RNA-seq from *Phaseolus vulgaris* available (February 2016) in the Sequence Read Archive were downloaded and small RNA-seq were filtered. A total of 157 libraries belonging to 105 different samples were selected for the AS analysis in common bean. Eighty four libraries belonging to 77 different samples from *Glycine max* were selected based on project or tissue similarity with those from *P. vulgaris*. (Additional file 9). All available EST from NCBI and TGI database [60] from common bean and soybean were analysed. EST’s from both databases were sequenced form multiple tissues and conditions. In total 176,782 and 1,461,723 EST sequences from common bean and soybean, respectively, were analysed.

### Mapping and AS annotation

All RNA-seq libraries were mapped to their respective genome without gene annotation information, alignments must be unique and perfect. For the mapping two different approaches were used; a seed and extend approach, where reads are sliced into short seeds, which are mapped to the genome, allowing the identification of splicing sites; and an exon first approach, where complete reads are mapped at first and based on that mapping an exon-exon junctions database is created in order to align the unmapped reads later [61]. TopHat2 [62] was used for the exon first approach and gsnap [63] for the seed extended (Fig. 2). Each mapping result, one for gsnap and one for TopHat2, was the input for a gene prediction modelling performed with Cufflinks [64], this was carried out also without any gene annotation information as well (Fig. 2). EST’s were mapped to their respective genome with Gmap [63] (Fig. 2). The gene models were filtered to avoid chimeras based on the coordinates of the primary transcript of each gene in the genome annotation. Models in zones where genes overlap or models that are part of two or more genes were removed. Each gene model from each mapping algorithm of each RNA-seq library was compared to their corresponding annotated primary transcript in the genome with an in-house perl script (Fig. 2). This algorithm identifies the seven different AS events (AA, AD, ASS, ES, IR, NI and RE) by comparing the genome coordinates of the out coming gene models to all primary transcripts (Fig. 1). AS events present in EST’s, or in both mapping results for a particular library, or in at least half of the sample replicates from a type of mapping were selected for further analysis (Fig. 2 and Additional file 2).

### Gene expression

The expression value for each gene was calculated using all FPKM values from each model that belong to each gene (Fig. 2). The FPKM from each model was multiplied by the length of the model, the sum of all products from each gene was then divided by the length of the primary transcript resulting in a normalized value of expression per mapping algorithm. The mean of both normalized expression value, one for gsnap and other for TopHat2, was the library expression. The sample expression was calculated by the median of the replicates libraries expressions values. A gene was considered expressed in a tissue if the sample expression was above one.

### RT-PCR analysis

For RNA isolation surface-sterilized seeds from *P. vulgaris* and *G. max* were germinated over moist paper, in sterile conditions, for 2 days. The root and cotyledonary leaves from germinated seedlings were cut, frozen in liquid nitrogen and stored separately at −80 C until used. Total RNA was isolated from 200 to 400 mg frozen tissue using Trizol reagent (Life Technologies, California, USA), as reported [65]. Absence of genomic DNA contamination was subsequently confirmed for each sample by PCR amplification using primers for the ACR9 (ACT-domain containing protein) gene (Phvul.008G013100, Glyma.18G289000). To validate the presence of different transcript isoforms identified through bioinformatics analysis, two-step RT-PCR was performed following the manufacturer’s directions (Thermo Scientific, USA) using poly-thymine deoxynucleotide primer. Eight genes with from *P. vulgaris* and their corresponding *G. max* homologs were selected for AS events validation and the ACR9 gene that did not present AS was included as a control. For each selected gene, a pair of oligonucleotide primers was designed to amplify products specific for the primary transcript or for transcript isoforms derived from AS events; primer sequences as well as genes IDs and annotation are shown in Additional file 10. For RT-PCR reactions the thermocycler was set to: 60 / 68 °C for annealing / extension and 35–40 cycles and a High Fidelity DNA Polymerase (Jena Bioscience, Germany) was used. Amplification products were resolved in a 3% agarose gel in 1xTAE and EtBr stained for visualization.

### AS simulation

Simulation of AS were performed based on number of exons/introns of expressed genes. Four different types of simulations were performed: “random events”, “random genes”, “random genes same model” and “random genes + random event”. One thousand independent simulations were performed for each type of simulation.

### AS event conservation

*Phaseolus vulgaris* and *Glycine max* orthologous genes were pulled out from Schmutz et al. [34]. There were 13,962 common bean genes with two orthologous genes in soybean, resulting from the recent whole genome duplication in this legume, and 5624 orthologs with only one copy in soybean. The AS conservation was based on junction conservation and not in position conservation. Due to this fact, genes should have the same gene model (same number of exons) and been expressed in at least one sample (7′692 common bean genes with two orthologous genes in soybean and 7′039 with one gene).

## Additional files

Additional file 1: AS events reported in the *Phaseolus vulgaris* [34] and *Glycine max* [33] annotated genomes. (XLSX 11 kb)

Additional file 2: AS event. (XLSX 8631 kb)

Additional file 3: Splicing sites. Percentage of splicing sites motifs reported in the genome (inner circle), in the new junctions (middle cirlce) and the genome with the new junctions (outer circle). U2 motifs (gray), U12 motifs (black) and non-canonical splicing sites (striped). Panel a show the results from *P. vulgaris* while b from *G. max*. (TIFF 816 kb)

Additional file 4: Introns and exons from CDS and UTR regions affected by AS events. Percentage of *P. vulgaris* and *G. max* introns (a) and exons (b) affected by AS compared to their total proportion in each genome. Proportions of common bean as well as soybean are plotted. (TIFF 159 kb)

Additional file 5: Percentage of non-redundant AS event types in homologous genes. (XLSX 11 kb)

Additional file 6: Four AS conservation simulations. Four different simulations for AS event conservation percentage testing were performed. “random events”: randomize the AS events in the expressed genes maintaining homologous genes; “random genes”: randomize homologous genes, the gene model was not taken into account but the events remained as the real data; “random genes same model”: same as “random genes” but the gene model stays equal and “random events + random genes”: AS events as well as homologous genes, ignoring real gene models, were randomized. (TIFF 232 kb)

Additional file 7: Exon distribution. Proportions of number of exons per gene in the annotated *P. vulgaris* and *G. max* genomes, homologous genes with an evolutionary relationship of with evolutionary relationship 1:2 and 1:1 and pseudo-homologous genes resulted from “random genes” simulation. (TIFF 115 kb)

Additional file 8: Comparison of conservation percentages of AS events in *Glycine max.* Data from each performed simulation are plotted with a different color while the blue line corresponds to the values of percentage of AS conservation shown in Fig. 6b. The percentage of AS conservation of *G. max* in *P. vulgaris* considered over all AS events in soybean were analyzed. For description of each plot see legend to Fig. 7. (TIFF 136 kb)

Additional file 9: Sample used for the AS event identification. (XLSX 41 kb)

Additional file 10: *P. vulgaris* and homologous *G. max* genes selected for RT-PCR analysis. (XLSX 9 kb)

## Abbreviations

AA: Alternative acceptor
AD: Alternative donor
AS: Alternative splicing
ASS: Alternative splicing sites
CDS: Coding DNA sequences
DN: Nonsynonymous mutations
DS: Synonymous mutations
ES: Exon skipping
EST: Expressed sequence tags
IR: Intron retention
MYA: Millions years ago
NI: New intron
NMD: Nonsense-mediated decay
RE: Retained exon
RNA-seq: Next generation RNA-sequencing
snRNA: Small nuclear RNAs
SR: Serine/arginine-rich proteins
uORF: Upstream open reading frame
UTR: Untranslated region
WGD: Whole genome duplication
