# Supplementary figures and images for: Identification and analysis of alternative splicing events in Phaseolus vulgaris and Glycine max
Source: BMC Genomics. 2017 Aug 22;18:650. doi: 10.1186/s12864-017-4054-2 (PMC5568362; doi:10.1186/s12864-017-4054-2)

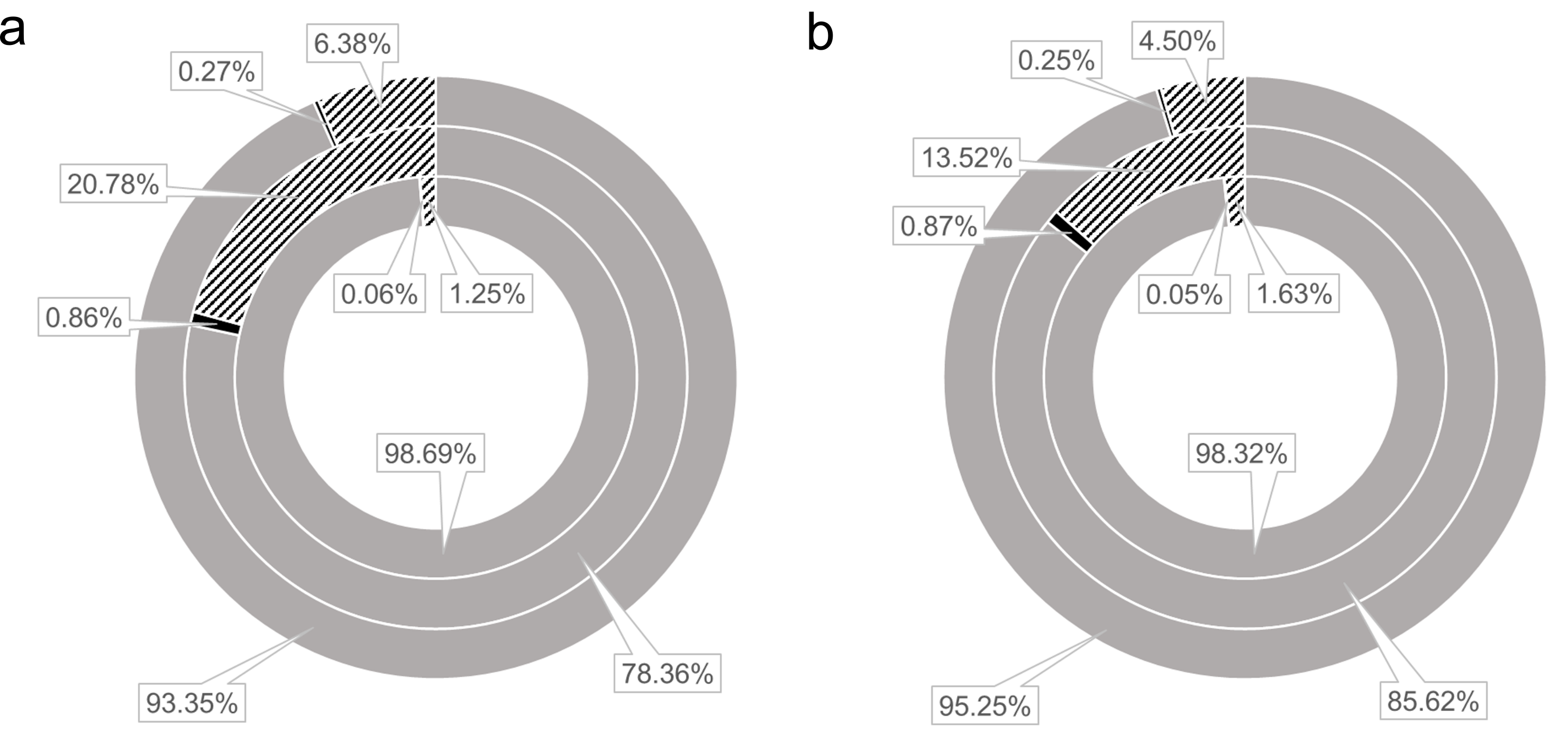

Supplement: Supplementary file 3 — Splicing sites. Percentage of splicing sites motifs reported in the genome (inner circle), in the new junctions (middle cirlce) and the genome with the new junctions (outer circle). U2 motifs (gray), U12 motifs (black) and non-canonical splicing sites (striped). Panel a show the results from P. vulgaris while b from G. max. (TIFF 816 kb) [file 12864_2017_4054_MOESM3_ESM.tif]

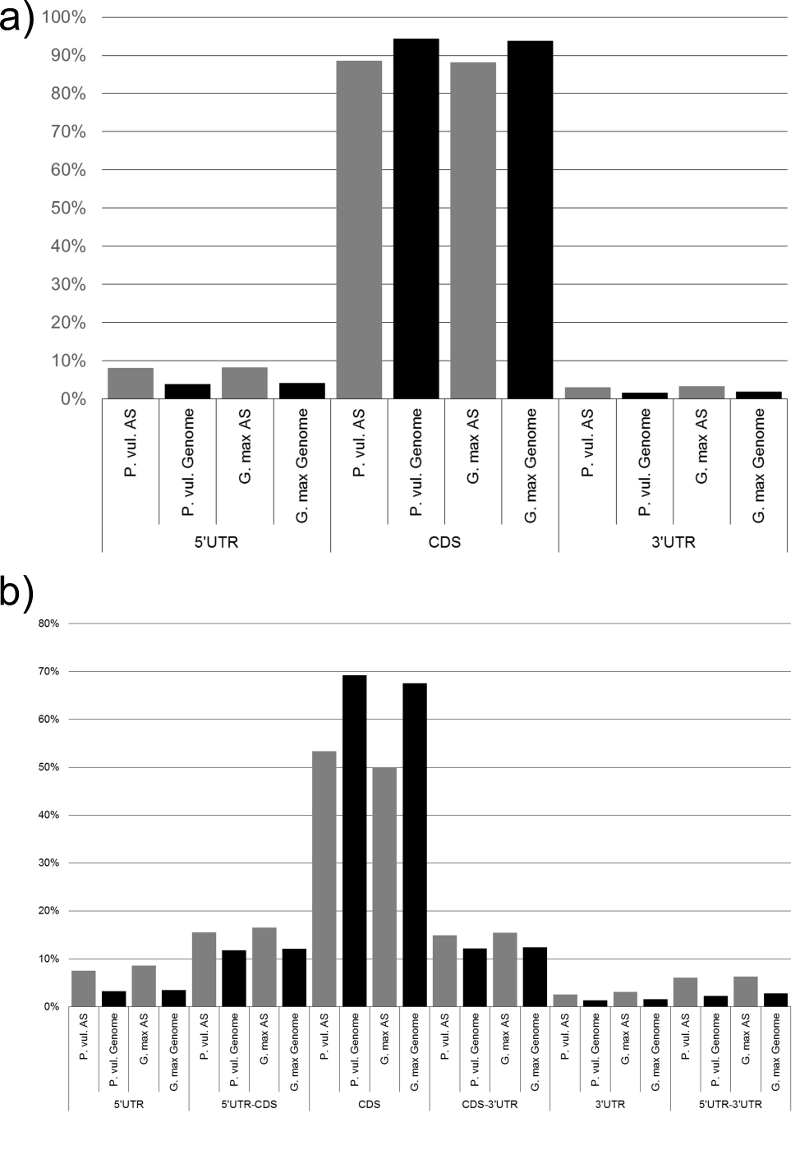

Supplement: Supplementary file 4 — Introns and exons from CDS and UTR regions affected by AS events. Percentage of P. vulgaris and G. max introns (a) and exons (b) affected by AS compared to their total proportion in each genome. Proportions of common bean as well as soybean are plotted. (TIFF 159 kb) [file 12864_2017_4054_MOESM4_ESM.tif]

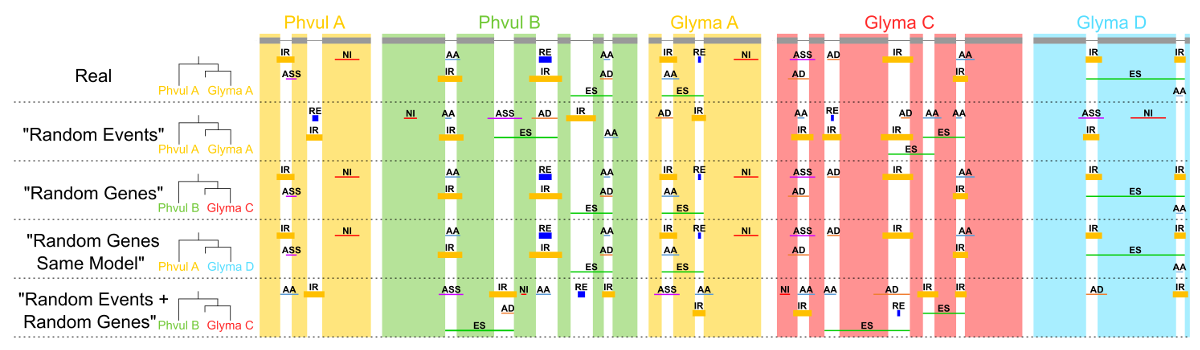

Supplement: Supplementary file 6 — Four AS conservation simulations. Four different simulations for AS event conservation percentage testing were performed. “random events”: randomize the AS events in the expressed genes maintaining homologous genes; “random genes”: randomize homologous genes, the gene model was not taken into account but the events remained as the real data; “random genes same model”: same as “random genes” but the gene model stays equal and “random events + random genes”: AS events as well as homologous genes, ignoring real gene models, were randomized. (TIFF 232 kb) [file 12864_2017_4054_MOESM6_ESM.tif]

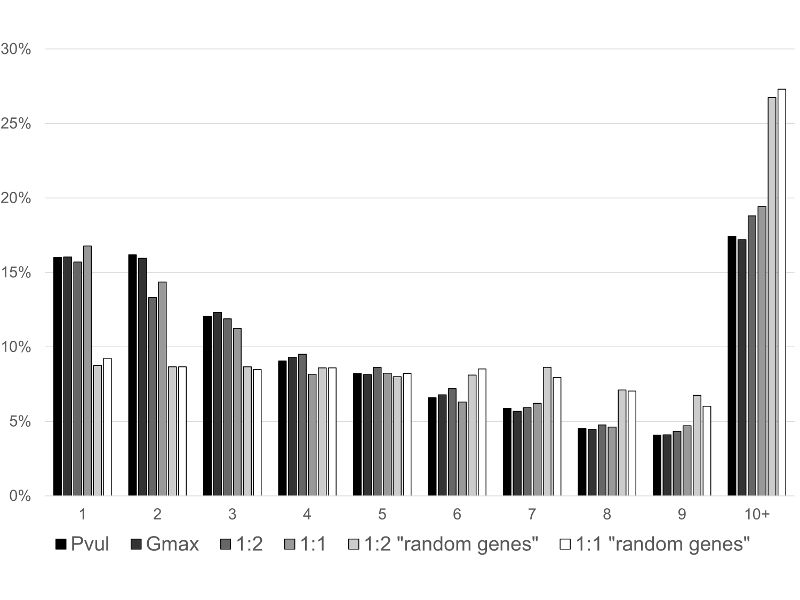

Supplement: Supplementary file 7 — Exon distribution. Proportions of number of exons per gene in the annotated P. vulgaris and G. max genomes, homologous genes with an evolutionary relationship of with evolutionary relationship 1:2 and 1:1 and pseudo-homologous genes resulted from “random genes” simulation. (TIFF 115 kb) [file 12864_2017_4054_MOESM7_ESM.tif]

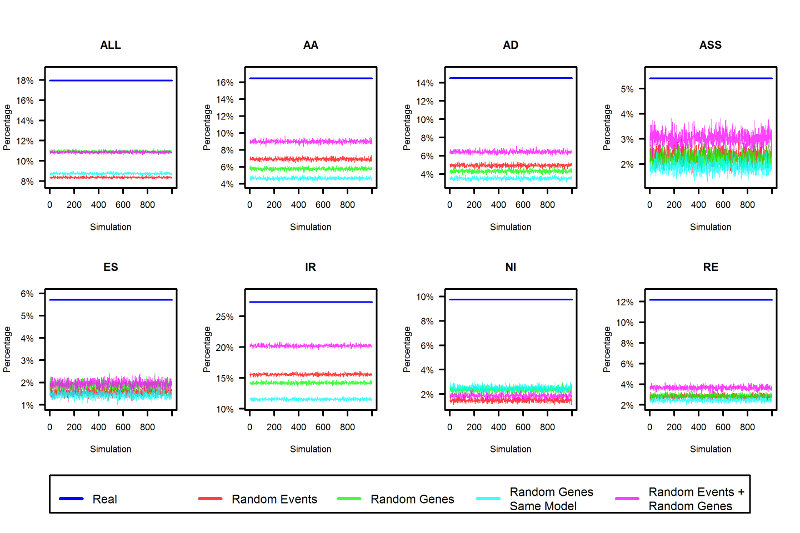

Supplement: Supplementary file 8 — Comparison of conservation percentages of AS events in Glycine max. Data from each performed simulation are plotted with a different color while the blue line corresponds to the values of percentage of AS conservation shown in Fig. 6b. The percentage of AS conservation of G. max in P. vulgaris considered over all AS events in soybean were analyzed. For description of each plot see legend to Fig. 7. (TIFF 136 kb) [file 12864_2017_4054_MOESM8_ESM.tif]
